# The Antidote to Carbolic Acid*Read before the Homœopathic Medical Society of the county of New York, February 8th, 1894.

**Published:** 1894-03

**Authors:** E. Carleton

**Affiliations:** New York


### THE ANTIDOTE TO CARBOLIC ACID.*

E. Carleton, M. D., New York.

It is well known to the medical profession that the action of Carbolic Acid is so destructive to the human body that if taken internally, even in small quantity, death speedily ensues. Every few days we read in the daily newspapers of some person

---

* Read before the Homœopathic Medical Society of the county of New York, February 8th, 1894.

who has, by design or accident, swallowed a few drops or more of this acid, and soon after died, in spite of all that physicians and chemists could do. Tragic indeed was the case of a physician who was standing in an apothecary's shop, chatting with his medical and pharmaceutical friends. Some whiskey in a tumbler had been placed upon the counter near him, which he intended to drink. But various other glasses stood upon the same counter also. The physician's face was turned toward his friends. He put his hand out mechanically for the whiskey, took a glass containing Carbolic Acid, raised it to his lips and swallowed a small portion of the poison. Dashing the glass from him he exclaimed, "I'm a dead man!" His friends did all they could for him; but in a few moments his words became verified.

No antidote to Carbolic Acid has been known to the profession at large. Let me now call your attention to an antidote that is simple, safe, and effectual, and so common as to be found ready for use in every household. Knowledge of this should be made public, as a means of averting many disasters. Homœopathic physicians will probably not see many cases of poisoning in the families that usually consult them. Lack of opportunities to try the antidote accounts for the tardy production of this paper. But we may sometimes be called to cases of this kind, and then should be equipped. And if our neighbors of the Old School will accept truth at our hands, it will be of great service to them and to the users of crude drugs who are accustomed to look to them for advice.

Like many other useful discoveries, this came by accident. I spoke of it to some of my colleagues; but naturally felt like waiting for more evidence before making a statement to any learned Society. Finally my conscience would not let me wait any longer, and I read the following brief account before the International Hahnemannian Association at Saratoga, N. Y., in June, 1886:

"CIDER VINEGAR AS A LOCAL ANTIDOTE TO CARBOLIC ACID.

"A few years ago, I was making laboratory experiments with pure Carbolic Acid, when, by an accident, about two ounces of the pure acid was suddenly dashed upon my hands. A stream of water followed as soon as it could be obtained, but it was useless. The usual result followed quickly—white skin and paralysis of the nerves of sensation wherever the acid had touched. Inasmuch as no antidote was known, I resolved to be a philosopher and let time work recovery. But the fumes of Carbolic Acid are very disagreeable to me; and there being some cider vinegar in the kitchen, that was suggested to my mind as likely to furnish a tolerable substitute in the way of odor. So upon the hands it went. To my amazement, the white color began to leave, and in a few minutes, by using vinegar freely, the natural color and function of the members were fully restored.

"This chemical action has since been verified upon others—upon the skin only, to my knowledge. Would it not be well to try it upon the mucous membrane, if occasion offers? I do not know of vinegar having been employed internally for this purpose. Some one will ask if the Acetic Acid of the shops has the same action. My answer is I do not know; I have not tried it."

So far as the skin is concerned, a great many verifications of the foregoing have since been noticed by myself and others. I have never had opportunity to use vinegar against Carbolic Acid on the mucous membrane; but Dr. C. Spencer Kinney, with whom many of you are personally acquainted, has sent the following interesting history:

December, 1893.

*Statement by C. Spencer Kinney, M. D., Hospital for the Insane, Middletown, N. Y.*

"At seven o'clock in the morning of August 4th, 1884, a nurse called me to see a man who had swallowed some Carbolic Acid. The patient was found with his lips, mouth, and tongue coated white where the acid had touched them, and the strong characteristic odor of the acid was present. He was at once given a half cup of vinegar diluted with an equal amount of water, and this was followed in a few moments by a second dose of vinegar and water. As the time hung heavily on my hands while waiting for the stomach-pump, the patient was given some milk, which he willingly drank. The odor and the discoloration from the acid had disappeared from the patient's lips, mouth, and tongue on taking the vinegar and water, and on using the stomach-pump no odor from the liquid that was pumped from his stomach could be detected. After the stomach had been carefully washed out the patient was fed with hot milk for several days, and no further symptoms developed.

"It was not until May, 1887, that I saw, in *The Homœopathic Recorder*, an article which had been presented before the International Hahnemannian Association, by Dr. Edmund Carleton, of New York, on the use of vinegar as an antidote. I have always thought I was indebted to him for the knowledge of this action of vinegar, as my acquaintance with Dr. Carleton antedated my use of vinegar as an antidote of Carbolic Acid by a number of years, and I may have heard it from him. Since seeking his explanation for the use of vinegar as an antidote for the acid, I have had an opportunity to test its efficiency in a number of instances, and have always found it to be reliable in every particular, and in no instance where the vinegar has been used within a few moments has there been any eschar formed."

This furnishes the desired evidence. I have now no hesitation in saying that cider vinegar is the antidote to Carbolic Acid.

